# The anti-cancer effects of Tualang honey in modulating breast carcinogenesis: an experimental animal study

**DOI:** 10.1186/s12906-017-1721-4

**Published:** 2017-04-11

**Authors:** Sarfraz Ahmed, Nor Hayati Othman

**Affiliations:** 1 Department of Pathology, School of Medical Sciences, Universiti Sains Malaysia, Kubang Kerian, Kelantan Malaysia; 2 Department of Biochemistry, Bahauddin Zakariya University, Multan, 60800 Pakistan

**Keywords:** Tualang honey, Experimental breast cancer, Anti-cancer effect, Estrogen receptor 1, Apaf-1, Honey effect on hematological Parameters

## Abstract

**Background:**

Honey has been shown to have anti-cancer effects, but the mechanism behind these effects is not fully understood. We investigated the role of Malaysian jungle Tualang honey (TH) in modulating the hematological parameters, estrogen, estrogen receptors (ER1) and pro and anti-apoptotic proteins expression in induced breast cancer in rats.

**Methods:**

Fifty nulliparous female Sprague–Dawley rats were used and grouped as follows: Group 0 (healthy normal rats control), Group 1 (negative control; untreated rats), Groups 2, 3 and 4 received daily doses of 0.2, 1.0 and 2.0 g/kg body weight of TH, respectively. The rats in groups 1, 2, 3, 4 were induced with 80 mg/kg of 1-methyl-1-nitrosourea (MNU). TH treatment in groups 2, 3 and 4 was started one week prior to tumor induction and continued for 120 days.

**Results:**

The TH-treated rats had tumors of different physical attributes compared to untreated negative control rats; the tumor progression (mean 75.3 days versus 51.5 days); the incidence (mean 76.6% versus 100%); the multiplicity (mean 2.5 versus 4 tumor masses per rat); the size of tumor mass (mean 0.41 cm versus 1.47 cm [*p* < 0.05]) and the weight of the tumor mass (mean 1.22 g versus 3.23 g; [*p* < 0.05]). Histological examinations revealed that cancers treated with TH were mainly of grades I and II compared with the non-treated control, in which the majority were of grade III (*p* < 0.05). TH treatment was found to modulate hematological parameters such as Hb, RBCs, PCV, MCV, RDW, MCHC, polymorphs and lymphocytes values. TH treatment groups were found to have a lower anti-apoptotic proteins (E2, ESR1 and Bcl-xL) expression and a higher pro-apoptotic proteins (Apaf-1 and Caspase-9) expression at serum and on cancer tissue level (*p* < 0.05).

**Conclusion:**

Tualang Honey alleviates breast carcinogenesis through modulation of hematologic, estrogenic and apoptotic activities in this experimental breast cancer animal model. Tualang Honey may be used as a natural ‘cancer-alleviating’ agent or as a supplement to chemotherapeutic agents.

## Background

Breast cancer, similar to other cancers, exhibits major morbidity and mortality. It is continued to be the most common female cancer and prevalence is still rising [1]. Cancer-prevention is aimed at interfering with the basic processes of carcinogenesis through chemical agents or regimens that may block neoplastic induction [2]. It results in prevention of the progression of transformed cells into malignant types [2]. The other purpose is to prevent the development of second primary tumors that may arise from patients who had already been cured from the initial cancer [2]. Potential cancer-preventive agents include vitamins, minerals, phytochemicals, synthetic compounds and natural products such as honey [3, 4].

Honey has been used since ancient times as a traditional medicine. Honey has recently received attention as a complementary and alternative treatment in modern medicine [4, 5]. It is mainly composed of various sugars, phenolic acids, flavonoids, enzymes, amino acids, proteins, phytochemicals and other miscellaneous compounds [4]. It is also considered as a natural phytoestrogen [6] with epigenetic modification [7]. Honey has a potential to be preventive agent against cancer [4]. Tualang honey (TH) is a multi-floral jungle honey. It is produced by *Apis dorsata* bee specie which builds their hives high on Tualang trees (*Kompassia excelsa*) in Malaysian tropical rainforests [8, 9]. Published data has shown that TH exhibits antimicrobial [10, 11], anti-inflammatory [9], antioxidant [12] and antidiabetic effects [13]. It has been demonstrated that TH as a cancer-preventive agent ameliorates 7,12-dimethyl-benz-anthracene (DMBA) induced breast cancer in vivo by modulating tumor severity, histological grading and increased apoptosis [14]. Another study has reported that TH reduces tamoxifen-induced cytotoxicity against breast cancer *in vitro*, demonstrating its preventive effects [15]. TH has been shown to have anticancer effects against oral squamous cell carcinoma [16], human osteosarcoma cell lines [16], human breast cancer cell lines [17] and cervical cancer cell lines [17]. The aim of our study was to evaluate the chemo-preventive effects of Tualang honey against experimental breast cancer in vivo.

Hematological parameters have been correlated with prognosis in different malignancies [18]. Pre and post-treatment studies have shown that breast cancer patients have deranged or abnormal blood count pattern [18]. The functioning of the immune system at hematological level has a direct influence on breast cancer [19]. Prolonged exposure to estrogens has been associated with increased risk of breast cancer development [20]. Elevated serum levels of endogenous estrogen (E2) and estrogen receptors (ESR1, ESR2) are associated with increased risks [21]. Estrogen receptor positive breast cancer is the most common type and anti-estrogen therapy has been shown to be very effective in preventing recurrence [22].

Apoptosis is recognized as the principal mechanism of drugs-induced regression in breast cancer [23]. The expression of pro and anti-apoptotic proteins is considered a hallmark for prognosis of this disease [23]. Apoptotic protease activating factor-1(Apaf-1) is a tumor suppressor gene [24]. A reduction in Apaf-1 occurs during tumor progression from primary to systemic metastasis and can contribute to the ability of tumor cells to evade Caspase-9 apoptotic pathway [24]. Increased levels of Bcl-xL expression are seen in primary high grade human breast carcinomas [25].

The beneficial ‘preventive’ effects of TH on breast cancer are based on the premise that there is a reduction of tumor incidence, increased latency period and a slower tumor growth through modulation of hematologic, estrogenic and apoptotic activities. To our knowledge, this is the first study to report the modification of hematological parameters, E2, ESR1 and Apaf-1 by administering honey as a preventive measure in an in-vivo breast cancer model.

## Methods

### Animals and honey

We used virgin Sprague–Dawley (SD) female rats aged between 28 and 35 days old obtained from Animal Research and Service Centre (ARASC), Universiti Sains Malaysia (USM), Kubang Kerian Kelantan, Malaysia. The experimental protocol used in this study was approved by the animal ethics committee of our institution [Reference number: USM//2011/(68)(306]. Tualang honey (TH) was supplied by Federal Agricultural Marketing Authority (FAMA), Ministry of Agriculture and Agro-based Industry, Malaysia. The honey samples were filtered, evaporated at 40 °C (to achieve 20% water content) and were subjected to gamma irradiation at 25 kGy for sterilization purposes (STERILE GAMA™, Selangor, Malaysia). Water content of the honey was measured using a digital ABBE refractometer (ATAGO CO., Japan). The refractive index values were converted to moisture contents [26]. The moisture content of honey is of critical importance as it affects the quality of honey and its resistance to microbial spoilage. Low water content is desired because honey begins to ferment if the water content is greater than 20% [27].

### Study design

A total of 50 female SD rats were divided into 5 groups with 10 animals per group. The rats were maintained on a standard balanced rat feed diet with water ad libitum and a 12 h day/night cycle. Group 0: healthy tumor free rats (normal rats control); Group 1: negative control (untreated tumor bearing rats); Group 2: rats receiving TH 0.2 g/kg body weight/day (low dose). Group 3: rats receiving TH 1.0 g/kg body weight/day (Medium dose); Group 4: rats receiving TH 2.0 g/kg body weight/day (high dose). Honey treatment by oral feeding (using 1 ml syringes without needles administered to mouth) was started 1 week prior to tumor induction for groups 2, 3 and 4. The rats in groups 1, 2, 3 and 4 were induced with cancer using carcinogen 1-methyl-1-nitrosourea (MNU). MNU (Catalog no. N1517-1G, Sigma, USA) was prepared in 0.9% NaCl solution acidified to pH 5.0 with 0.05% acetic acid, as previously described [28]. In total, 80 mg/kg body weight of MNU was injected intra-peritoneally (i.p.) when the rats were 40 days old. Honey treatment was continued for 120 days for rats in groups 2, 3 and 4. The rats’ breasts were palpated twice weekly to detect the appearance and progression of tumor masses. The incidence (the percentage (%) of tumor-bearing rats in the group), the latency (the number of days taken for the rats to develop first tumor mass) and the size (in cm^3^) of the tumor masses were recorded. Tumor size was measured using an established formula *Tumor size* = 1/2(*length* × *width* ^2^) [29]. On the 120th day of treatment, rats were subjected to necropsy after i.p injection of pentobarbital 100 mg/kg body weight. Blood samples were collected by cardiac puncture into EDTA tubes for hematological parameters and some portion was placed in plain tubes for serum separation. Tumor masses were examined in vivo prior to excision. Each tumor mass was fixed in neutral buffered formalin for histological and immunohistochemical analysis. Blood samples in plain tubes were left to clot for 2 h prior to centrifugation for 15 min at 4000 rpm (Eppendorf centrifuge, Germany). Approximately 1 ml of serum was collected and stored at −80 °C until assayed.

### Determination of body weight

The total body weight of rats was measured using a digital analytical balance (Sartorius AG, Germany) weekly from start of treatment till end of study. The percentage body weight changes were calculated at the end of study (week 16). The actual body weight changes were calculated by subtracting the weight of tumors at week 16. The formula used to calculate percentage weight gain is described as follows [14];

$$ \mathrm{Percentage}\ \mathrm{body}\ \mathrm{weight}\ \mathrm{change}\ \mathrm{or}\ \mathrm{gain}\ \left(\mathrm{BW}\ \mathrm{change}\%\right)=\left[\left(\mathrm{FBW}\hbox{--} \mathrm{IBW}\right)\times 100\right]/\mathrm{IBW} $$

$$ \mathrm{Actual}\ \mathrm{body}\ \mathrm{weight}=\mathrm{Body}\ \mathrm{weight}\ \mathrm{at}\ \mathrm{week}\ 16\hbox{--} \mathrm{weight}\ \mathrm{of}\ \mathrm{tumors} $$

$$ \mathrm{Percentage}\ \mathrm{actual}\ \mathrm{body}\ \mathrm{weight}\ \mathrm{change}\ \mathrm{or}\ \mathrm{gain}\ \left(\mathrm{ABW}\ \mathrm{change}\%\right)=\left[\left(\mathrm{ABW}\hbox{--} \mathrm{IBW}\right)\times 100\right]/\mathrm{IBW} $$

### Determination of hematological profile

Full blood count (FBC) was carried out using an automated cell count analyzer (Sysmex KX-21, Japan). Auto analyzer was capable to run several parameters for each sample such as hemoglobin concentration (Hb), packed cell volume (PCV), red blood cell (RBC), red blood cells distribution width (RDW), mean cell volume (MCV), mean corpuscular hemoglobin (MCH), mean corpuscular hemoglobin concentration (MCHC), platelet, total white blood cell counts (TWBC), polymorphs, lymphocytes, monocytes, eosinophils and basophils. The equipment sampling probe aspirated 20 μl well mixed blood samples and the result of analysis was obtained accordingly. A total of eight to nine samples were run per batch.

### Histopathological examination of the breast cancer masses

Formalin-fixed tumors were sectioned (3 μm thickness), mounted on frosted-end glass slides, deparaffinized and stained with hematoxylin and eosin using the standard method. The stained sections were examined under a light microscope at 100×, 200× and 400× magnification using an Olympus BX41 microscope (Olympus Optical Co. Ltd., Tokyo, Japan). The tissue sections were examined by pathologist (NHO) who was blinded to the treatment groups. The tumors were graded with a human cancer grading system using the modified Bloom and Richardson technique [30].

### Immunohistochemical analysis for pro- and anti-apoptotic proteins in cancer tissue masses

Tissue blocks were sectioned at 3 μm and immunohistochemically stained for Caspase-9 Rabbit polyclonal Mouse Anti-Rat Caspase-9 Antigen (Catalog no. GTX73093, Inc., GeneTex, USA; diluted at 1:25), Apaf-1 with Mouse monoclonal Anti-Rat Apaf-1 Antigen (Catalog no. SC-65891, Inc., Santa Cruz Biotechnology, USA; diluted at 1:100), FASLG with monoclonal Mouse Anti-Rat FASLG Antigen (Catalog no PAB 8018, Inc., Abnova, Taiwan; diluted at 1:200), FADD with Rabbit polyclonal Anti-Rat FADD Antigen (Catalog no. GTX73104, Inc., GeneTex, USA; diluted at 1:25), ESR1 with polyclonal Rabbit Anti-Rat ESR1 Antigen (Catalog no. PAB 18170, Inc., Abnova, Taiwan; diluted at 1:100) and Bcl-xL with mouse monoclonal Anti-Rat Bcl-xL Antigen (Catalog no. MS-1334-P1, Inc., Labvision, USA; diluted at 1:100). The staining procedure was performed according to the manufacturer’s instructions. The expression of the proteins was assessed using a semi-quantitative scoring system developed by Allred and colleagues [31]. The positively stained cells were counted in a given area of each tissue on 10 fields by SA and verified by pathologist (NHO) in a blinded manner and the data were presented as a percentage of positivity.

### Determination of E2 and Apaf-1 at serum level

The serum levels of E2 and Apaf-1 were determined using a 50 μl serum sample with an E2 ELISA kit (Catalog no. CSB-E05110r, Inc., COSMO BIO, USA) and an Apaf-1 ELISA kit (Catalog no. BG-RAT10190, Inc., Novatein Bio Sciences, USA). Seven to eight serum samples per treatment and control groups were analyzed against known standards and a serum blank. The ELISA procedure was performed according to the manufacturer instructions. The results were obtained by calculating the mean absorbance at 450 nm (Spectrophotometer, Thermo Fisher Scientific Inc., Waltham, MA, USA) for each of the duplicate standards, controls and samples as stated by the manufacturer.

#### Statistical analyses

The data were analyzed using the program IBM SPSS, Statistics version 22. The Fisher Exact test was used to analyze tumor incidence, latency and grading. A mixed model two way repeated measures of ANOVA was conducted to evaluate the effect of treatments on rats body weight gain. A comparison of the median values between groups was performed by Kruskal-Wallis H test. The differences between two groups were identified by the Mann–Whitney U test followed by Bonferroni’s correction. Level of significance was set at *p* < 0.05.

## Results

### Tumor incidence, latency, size and weight

Palpable tumors were evident in all rats exposed to carcinogen (MNU). The subjects in the honey treatment groups (Groups 2, 3 and 4) exhibited a significantly lower tumor incidence (*p* < 0.05) and a higher latency period compared with the animals in the untreated tumor bearing negative control (Group 1). The differences among all TH treatment groups were not statistically significant. Similarly, the rats receiving various dosages of TH displayed a lower tumor multiplicity (*p* < 0.05), size and weight (*p* < 0.05) compared with untreated tumor bearing negative control. The details of tumor incidence, latency, multiplicity, size and weight in TH treated groups are shown in Table 1.

**Table 1:** The tumor parameters in TH treated groups compared with untreated tumor bearing negative control

Groups					
Tumor	1 -ive control	2 (0.2 g/kg TH)	3 (1.0 g/kg TH)	4 (2.0 g/kg TH)	*p* value
^a^Incidence (%)	100	80	80	70	0.406
^b^ Latency (days)	51.5 (14.75)	75.5 (29.75)	76.5 (19.25)	74 (23)	0.015
^b^ Multiplicity	4 (2.25)	2.5 (2.75)	3 (2.5)	2 (2)	0.190
^b^ Size (cm^3^)	1.47 (2.78)	0.26 (0.86)	0.38 (1.48)	0.60 (1.297)	0.000
^b^ Weight (g)	3.23 (7.23)	1.23 (5.23)	1.17 (2.50)	1.27 (2.97)	0.005

^a^Fisher Exact test. Values are statistically significant when *p* ≤ 0.05

^b^Kruskal-Wallis test. The data are expressed as median interquartile range (IqR) values. Values are statistically significant when *p* ≤ 0.05. ^a^Tumor incidence = the percentage of tumor-bearing rats in the group; ^b^Tumor latency = the duration in days for the first palpable tumor to develop after MNU administration; ^b^Multiplicity = No. of tumors developed. –ve control is untreated tumor bearing negative control

### Tumor progression

**Fig. 1:**
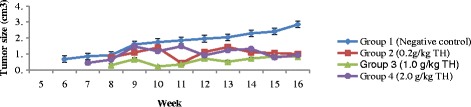
Tumor size (cm^3^) progression measured against time taken (in weeks) after MNU induction. Data are expressed as median interquartile range (IqR). Kruskal-Wallis test: values are not statistically significant in all weeks of experimental period, *p* > 0.05. Legends: TH = Tualang honey, MNU = 1-methyl-1-nitrosourea Negative control = group that received tumor induction but no honey treatment

The size of the tumors measured weekly during experimental period of 16 weeks showed that the tumors in TH treatment groups (Groups 2, 3 and 4) had a slower size increment and lesser median tumor size (<1.48cm^3^) compared to the untreated negative control group (Group 1) which showed a rapid size increment over shorter time period with the largest median size up to 2.85cm^3^ (*p* > 0.05). The difference is significant. The difference of tumor progression within varying dosages of TH among themselves was not significant (*p* > 0.05). Few of the tumors in TH treated-groups had regressed to non-palpable state. See Fig. 1.

### Body weight measurement

**Fig. 2:**
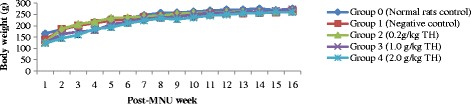
Body weight (g) progression over time (in weeks) after MNU induction. Data is presented as mean ± SEM. A mixed model two-way repeated measures ANOVA was conducted. A significant positive body weight progression was observed over time, (*p* < 0.05). Legends: TH = Tualang honey, MNU = 1-methyl-1-nitrosourea, Negative control = Group that received tumor induction but no honey treatment

In general, body weight of the rats in all groups (honey treated-groups & non-treated control groups) gradually increased throughout the experimental period over time (Fig. 2). Data for median body weight of rats in each group is presented in Table 2. At week 1, a significant difference in the median body weight between the groups was observed (*p* = 0.000). The median body weights in all treatment groups were lower compared to the rats of normal and negative controls (*p* < 0.05). At week 16, no statistical significant differences of median body weight between controls (negative and normal) and all treatment groups were observed. However, the rats of normal control group showed a slightly higher median body weight, BW change %, ABW, ABW% than all other groups. The percentage of change in body weight (BW change %) between the different groups was also statistically not significant. All treatment groups of TH presented a higher body BW change %, ABW, ABW% than untreated negative control group (Table 2).

**Table 2:** Body weight measurements of rats at week 1 and week 16

	Groups					
Body weight	0 Normal control	1 -ive control	2 (0.2 g/kg TH)	3 (1.0 g/kg TH)	4 (2.0 g/kg TH)	*p* value^a^
BW at week 1	168 (31.5)	139.5 (14)	122 (24.5)	123 (12)	120 (26.5)	0.000
BW at week 16	275 (29)	265 (36.25)	265.5 (25)	264.5 (38)	262.5 (45.75)	0.629
BW change (%)	119.04 (30.55)	94.66 (59.79)	118.01 (38.34)	117.84 (28.92)	118.15 (41.73)	0.275
ABW at week 16	275 (29)	245.55 (22.07)	264.43 (13.83)	263.18 (27.6)	256.28 (50.86)	0.008
ABW change (%)	119.04 (30.55)	73.94 (45.79)	114.935 (41.57)	117 (26.14)	115.47 (43.93)	0.018

^a^Kruskal-Wallis test: Data are expressed as median interquartile range (IqR). Values are statistically significant when *p* ≤ 0.05

### Macroscopic and microscopic evaluation

**Fig. 3:**
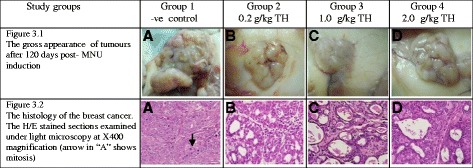
The gross morphology and histology of the breast tumors of rats in various TH-treated groups compared to the untreated negative control. The H/E stained sections were examined under light microscopy at X400 magnification. The majority of tumors in untreated negative control group were of grade III with increased heterogeneous nuclei formation and mitotic activity (plate A, arrow) compared to tumors in TH treatment groups which were of grade I and II (less aggressive) (Fig. 3.2); A = −ve control, B = 0.2 g/kg TH, C = 1.0 g/kg TH and D = 2.0 g/kg TH. Legends: TH = Tualang honey, MNU = 1-methyl-1-nitrosourea, −ve = Negative control (group that received tumor induction but no honey treatment)

Tumor masses in the non-treated negative control (Group 1) were larger in size, were solid and hard in consistency and exhibited areas of necrosis and hemorrhage. The tumors in groups 2, 3 and 4 were softer, paler and smaller in size (representative photographs in Fig. 3a). There were some tumor masses which completely shrunk during the study period. Histologically, the tumors from the negative control group were plump and showed a number of mitoses, while those in honey-treated groups were smaller and exhibited many degenerative cystic changes (representative histology in Fig. 3b). The majority of tumors in untreated rats were of higher histological grading (grade III) than tumors in honey-treated rats (grade I and II) (Table 3, Fig. 3b). In all groups, the majority of the tumors were found to be adenocarcinomas. Tumors in the negative control group were observed to have increased heterogeneous nuclei formation which were hyperchromatic, vesicular and highly pleomorphic with moderate cytoplasm and increased mitotic activity (more aggressive) compared to TH treatment groups which showed fatty change with small nucleus and cystic spaces (less aggressive) (Fig. 3b).

**Table 3:** Histological grading^b^ of tumors in various groups

Groups				
Tumor	1 -ve control	2 (0.2 g/kg TH)	3 (1.0 g/kg TH)	4 (2.0 g/kg TH)
Total No.	39	18	22	17
^a^Grade I (%)	7 (17.94)	11 (61.11)	9 (40.90)	9 (52.94)
^a^Grade II (%)	10 (25.64)	2 (11.11)	9 (40.90)	6 (35.29)
^a^Grade III (%)	22 (56.41)	5 (27.77)	4 (18.18)	2 (11.76)

^a^Fisher Exact test: Statistically significant difference between groups, *p* < 0.05. ^b^Modified Bloom Richardson Grading System. Legends: *TH* Tualang Honey, *−ve control* untreated negative control group

### Hematological parameters

The hematological values of the normal control rats were taken as the reference values as they were essentially normal rats. In general the TH-treated groups showed values closer to the normal control rats. The rats of the untreated tumor bearing negative control had a lower level of RBC [6.35 (0.75) 10^12^/L], Hb [14.1 (1.62) (g/dl], PCV [42 (3.25) %], lymphocytes [54 (20.75) %] and platelets [627.5 (196.75) 10^9^/L] compared to the rats of TH treated groups [RBC for 0.2 g/kg TH = 7.35 (1.22); 1.0 g/kg TH = 7.4 (1.025); 2.0 g/kg TH = 6.85 (1.67)], [Hb for 0.2 g/kg TH = 14.8 (1.92); 1.0 g/kg TH = 15 (1.97); 2.0 g/kg TH = 15.25 (2.77)], [PCV for 0.2 g/kg TH = 48.5 (5); 1.0 g/kg TH = 48.5 (9); 2.0 g/kg TH = 47.5 (8.75)], [Lymphocytes for 0.2 g/kg TH = 65 (13.5); 1.0 g/kg TH = 64.5 (13.5); 2.0 g/kg TH = 66.5 (15)], [platelets for 0.2 g/kg TH = 734 (197); 1.0 g/kg TH = 758.5 (178); 2.0 g/kg TH = 710 (89.5)]. While, TH-treated groups showed a lower level of TWBC, RDW, polymorphs and monocytes compared to the untreated negative control. There is significant difference between TH-treated and non-treated negative control for PCV, MCV, RDW, MCHC, polymorphs and lymphocytes values. The difference between all TH treatment groups among themselves was not statistically significant (Table 4). Overall, varying strengths of TH showed a potentiating effect on RBC, Hb, PCV, lymphocytes and platelets, while, a lowering effect on TWBC, RDW, polymorphs and monocytes compared to the untreated negative control (Table 4).

**Table 4:** The hematological parameters of TH-treated groups compared to controls

	Groups					
	0 Normal control	1 -ve control	2 (0.2 g/kg TH)	3 (1.0 g/kg TH)	4 (2.0 g/kg TH)	*p* value^a^
RBC (10^12^/L)	7.15 (0.27)	6.35 (0.75)	7.35 (1.22)	7.4 (1.025)	6.85 (1.67)	0.088
Hb (g/dl)	15.35 (0.62)	14.1 (1.62)	14.8 (1.92)	15 (1.97)	15.25 (2.77)	0.062
PCV (%)	48 (2.5)	42 (3.25)	48.5 (5)	48.5 (9)	47.5 (8.75)	0.047
MCV (fl)	65.5 (1.5)	66 (4.75)	65.5 (6.75)	66 (1.75)	65.5 (3.75)	0.004
MCH (pg)	21 (2)	21.5 (1.5)	20.5 (2.25)	21 (2.25)	21.5 (2)	0.958
MCHC (g/L)	32.5 (1)	31.5 (1.75)	31 (1.5)	31 (2.5)	32 (3)	0.000
RDW (%)	11.85 (1.7)	13.85 (1.7)	12.25 (2.725)	12.6 (1.5)	12.9 (2.22)	0.010
TWBC (10^9^/L)	4.85 (1.75)	6.14 (8.72)	5.05 (2.4)	4.95 (6)	6.25 (5.7)	0.178
Polymorphs (%)	33 (9.5)	42 (19.75)	34 (16.5)	32 (12)	32.5 (13.5)	0.009
Lymphocytes (%)	66 (5.5)	54 (20.75)	65 (13.5)	64.5 (13.5)	66.5 (15)	0.010
Monocytes (%)	1.5 (1.5)	1.5 (2.5)	1.25 (1)	1 (0)	1 (2)	0.649
Eosinophils (%)	0 (1.25)	0 (1.25)	0	0 (2)	0.5 (1)	0.534
Basophils (%)	0	0	0	0	0	1.000
Platelets’ (10^9^/L)	809.5 (149)	627.5 (196.75)	734 (197)	758.5 (178)	710 (89.5)	0.042

^a^Kruskal-Wallis test. Data are expressed as median interquartile range (IqR). Values are statistically significant at *p* ≤ 0.05. Legends: TH = Tualang honey, −ve = Negative (group received tumor induction but no honey treatment)

### Serum concentration of E2 and Apaf-1

**Fig. 4:**
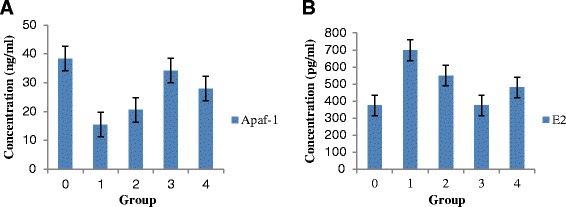
The serum level concentration of Apaf-1 (ng/ml) (Fig. 4**a**) and E2 (pg/ml) (Fig. 4**b**) in the rats of TH treated groups compared to the rats of normal and negative controls. Data are expressed as median interquartile range (IqR) using Kruskal-Wallis test. Values are statistically significant, *p* < 0.05. Legends: TH = Tualang Honey. Apaf-1 = Apoptotic protease activating factor-1. E2 = Estradiol

Serum levels of E2 and Apaf-1 in the normal control group (Group 0) were used as the reference range as they were essentially normal rats. Rats in the untreated negative control group (Group 1) expressed higher levels of E2 and lower levels of Apaf-1 compared with normal control rats. All the rats in the honey treated groups (groups 2,3 and 4) had levels of E2 and Apaf-1 approaching normal control values (Fig. 4a and b). The rats of un-treated negative control group had the least Apaf-1 median concentration (14.8, IqR 4.72 ng/ml) compared to those in TH treated groups followed by 0.2 g/kg TH (36.12, IqR 6.82 ng/ml), 1.0 g/kg TH (35.22, IqR 13.45 ng/ml) and 2.0 g/kg TH (35.83, IqR 2.90 ng/ml). The rats of the un-treated negative control presented a higher E2 concentration (697.44, IqR 113.01 pg/ml) compared to all those in TH treated groups followed by 0.2 g/kg TH (433.81, IqR 291.06 pg/ml), 1.0 g/kg TH (384.08, IqR 251.95 pg/ml) and 2.0 g/kg TH (506.43, IqR 157.61 pg/ml). The difference between the treated and non-treated groups was statistically significant (*p* < 0.05). The differences among treatment groups were not statistically significant (*p* > 0.05).

### Immunohistochemical expression of pro- and anti-apoptotic proteins

**Fig. 5:**
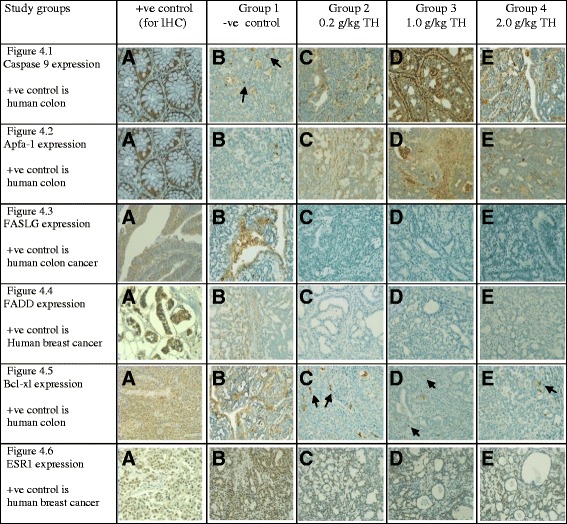
The immunohistochemical (IHC) expression of pro-apoptotic proteins; Caspase 9, Apaf-1, FASLG, FADD and anti-apoptotic proteins; Bcl-xl, ESR1 in TH treated tumors compared to the tumors of untreated control. A = +ve control for immunohistochemistry (IHC) analysis, B = −ve control for study (untreated tumor bearing rats), C = 0.2 g/kg TH, D = 1.0 g/kg TH and E = 2.0 g/kg TH. All specimens were examined at X400 microscopic magnification and brown color show antibody positivity. FASLG and FADD showed no expression in tumors of all treatment groups, while tumors in treated groups showed higher expression of pro-apoptotic proteins than those of non-treated control. Legend: TH = Tualang honey

The majority of tumors in the negative control rats had a higher percentage of immunohistochemical expression of ESR1 and Bcl-xL, and a lower expression of Caspase-9 and Apaf-1 compared with TH treated groups (Table 5). Representative immunohistochemical images are shown in Fig. 5. Tumors treated with TH showed no expression of FASLG and FADD (Table 5, Fig. 5). A significant statistical difference was observed between treated and non-treated groups (*p* < 0.05). The differences among honey treatment groups were not statistically significant.

**Table 5:** Immunohistochemical expression of pro- and anti-apoptotic proteins in breast tumors treated and non-treated with Tualang honey

Groups					
Tumors	1 -ve control	2 (0.2 g/kg TH)	3 (1.0 g/kg TH)	4 (2.0 g/kg TH)	S.E
Total No of tumors developed.	35	16	20	15	
No. of Caspase-9 positive tumors (% expression)	16 (45.7%)	13 (81.3%)	14 (70.0%)	11 (73.3%)	1.20
No. of Apaf-1 positive tumors (% expression)	15 (42.9%)	15 (93.8%)	16 (80.0%)	11 (73.3%)	1.28
No. of FASLG positive tumors (% expression)	13 (37.1%)	0	0	0	3.75
No. of FADD positive tumors (% expression)	12 (34.3%)	0	0	0	3.64
No. of Bcl-xL positive tumors (% expression)	28 (80.0%)	9 (56.3%)	10 (50.0%)	10 (66.7%)	5.29
No. of ESR1 positive tumors (% expression)	26 (74.28%)	8 (50%)	11 (55%)	9 (60%)	4.86

Kruskal-Wallis test; statistically significant differences between the groups, *p* < 0.05. Legend: *TH* Tualang Honey

## Discussion

The cancer-related death toll remains one of the highest among chronic diseases in humans [32]. A major concern for anti-cancer drugs is their potential toxicity [3]. Thus, alternative measures continue to be exerted to identify natural products with potential to complement existing preventive and therapeutic modalities [4]. Studies have shown the potential effectiveness of honey against cancer [4, 17]. Our study highlights some intriguing findings regarding the utility of TH as a potential cancer-preventive agent.

The findings of our study show that all the treatment groups that received TH had a lower tumor incidence and a higher latency than the non-treated positive control (Table 1). One of the primary purposes for cancer-preventive studies is to delay cancer appearance in healthy subjects or in subjects with an increased risk of cancer development who are otherwise healthy [33]. Thus, our findings suggest that tumor incidence was reduced and tumor initiation was delayed by TH treatment. Honey exhibits strong anti-oxidant and anti-mutagenic activity [9, 34], which could probably inhibit the carcinogenesis to transform the normal cells into malignant ones. Hence, may affect tumor latency and incidence.

The results of our study demonstrate that all varying strengths of TH appeared to slow down the progression of breast tumors development with lower multiplicity, size and weight compared to control (Fig. 1 and Table 1). It was also evident by the macroscopic evaluations of the tumors (Fig. 2). TH seems to be capable of reversing the tumorigenesis. It is shown by the reduced tumor size and weight in treated groups. Carcinogenesis is a multistep process and can be divided into three main stages; initiation, promotion and progression [35]. Cancer-preventive agents may act as anti-promoting agents via intervening at initiation or promotion stages of carcinogenesis. Thus, we can assume that TH may intervene at the initiation or promotion stage to inhibit tumor growth. Honey, which exhibits strong anti-oxidant and anti-mutagenic activity [9, 34], could potentially inhibit or disrupt critical steps in carcinogenesis, the initiation or promotion stage to inhibit tumor growth. The lower tumor multiplicity observed implies that TH may also acts as an anti-metastatic agent.

Some of the breast lesions in our study were found to be completely disappeared at the end of the study. It has been demonstrated that tumors can be eliminated or diminished by chronic administration of low doses of chemotherapeutic drugs [36]. It is quite possible that TH treatments behaves similarly. Our findings are consistent with another previous in vivo study investigated by Kadir and colleagues that honey may modulate tumor latency, incidence, multiplicity and progression [14]. In that study, a different carcinogen DMBA (7,12-dimethylbenzanthracene) was used. MNU has several advantages as it is more organ specific (breasts) and it induces tumors of breast ductal epithelium [28].

Histological grading of cancers has pivotal importance for the prognosis of the cancer [37]. The three criteria of grading breast cancer are based on the scores for mitotic activity, tubular formation by cancer cells and cellular pleomorphism. Our study shows that the breast cancers that developed in the TH treatment groups were of grades I and II, whereas tumors in the negative control group were mainly of grade III (Table 3 and Fig. 3). These findings support the antimutagenic activity of TH as it has been reported that honey exhibits antimutagenic activity [34]. Polyphenols and flavonoids are reported to be solely responsible for the anticancer activity of honey [38]. Thus, anti-neoplastic or anti-tumoral effects of TH may also be attributed to these compounds. Thus far, the specific compounds which are responsible for these protective effects are still unknown.

Full blood count is a prerequisite investigation in cancer patients before treatment and poor blood parameters affect the outcome of malignancies [18]. It has been demonstrated that hematological parameters are correlated with prognosis of cancer [18]. Breast cancer patients have been observed with abnormal or poor blood parameters [18]. Our results showed the TH-treated groups had blood parameters closer to the normal control rats. The untreated negative control rats had a lower level of RBC, Hb, PCV, lymphocytes and platelets compared to the rats of TH treated groups. Treatments with varying strengths of TH had potentiating effect on the hematological parameters such as RBC, Hb, PCV, lymphocytes, eosinophils and platelets (Table 4). Lymphocytes and eosinophils are cells of the immune system and good level of Hb could aid in recovery from the injury due to cancer. Cancer patients are reported to have lower level of RBC, Hb, MCV, MCH, MCHC and lymphocytes and higher level of RDW, TWBC, polymorphs during pre and post-treatment [18, 39]. Our findings show that TH may alter or tend to normalize the hematological parameters to ameliorate carcinogenesis in induced breast cancer. Exclusive honey feeding in the absence of any disease have been shown to significantly modify hematological parameters [40–43].

We noted that rats in negative control group had higher serum levels of E2 and a lower Apaf-1 concentration than TH treated rats. When given various strengths of honey (groups 2, 3 and 4), the levels of E2 and Apaf-1 tend to almost near normal levels (Fig. 4). Higher serum estradiol (E2) levels are associated with an increased risk of breast cancer in postmenopausal women [44]. The risk is higher in postmenopausal women and lower in premenopausal women [44]. Higher levels of E2 have been reported in breast cancer patients [21]. E2 promotes cell proliferation and suppresses apoptosis by directly modulating the genetic expression and thus is considered a crucial target in breast cancer treatment [45]. The reduction of E2 in TH treated groups could be attributed to honey. Treatment with estrogen-lowering drugs shrinks breast cancer masses in patients [46]. Thus, TH behaves as a natural estrogen-lowering agent.

At the tissue level, majority of tumors from the negative control group demonstrated a higher ESR1 expression than treated groups as detected by immunohistochemistry (Table 5 and Fig. 5). These tissue findings are validated when we found same results for E2 at serum level. Breast cancer patients had higher levels of estrogens and ER-mediated bioactivity [21]. Estrogen receptors (ERs) bind to estrogens to dimerize and then translocate into the nuclei. These complexes then bind to specific DNA base sequences called estrogen-response elements (EREs) resulting in transcription and translation in the targeted tissue [47]. This signaling cascade induced by estrogens may be modulated at any stage [47]. TH possibly modulates E2 and ESR1, hindering this signaling pathway. Exogenous or synthetic estradiol (E2) can be used as a treatment in estrogen receptor (ER) positive breast cancer to stimulate the apoptotic pathway [48]. Honey, which is a natural phytoestrogen [6], may play a role in modulating endogenous estrogen and estrogen receptors and may stimulate the apoptotic pathway.

TH may bind to the ER in a similar manner as other drugs that disrupt receptor dimerization to block ER nuclear localization [49]. Honeys from various floral sources exert estrogen agonist effects at high concentrations (20–100 μg/mL) and antagonistic effects at low concentrations (0.2–5 μg/mL) [50]. These effects were attributed to polyphenols or flavonoids content [50]. This study reported in vitro analysis of estrogen receptors only. Our study reports in vivo analysis of serum level estrogen and estrogen receptors at tumor tissues level. Our findings demonstrate agonist effect with all varying strengths of TH. The medium dose and low dose of TH seem more effective to modulate E2 and ESR1 respectively.

Loss of pro-apoptotic protein Apaf-1 can aid tumor cells in evading programmed cell death or apoptosis [51]. Apaf-1 is an essential target in the intrinsic or Caspase-9 apoptotic pathway [51]. Our results show TH has a potentiating effect on Apaf-1 and Caspase-9 at cancer tissues level (Fig. 4). This finding is further substantiated for Apaf-1 at serum level (Table 5 and Fig. 5). It could be concluded that TH enhances the expression of Caspase-9 and Apaf-1 resulting in slower tumor growth rate and better histological grading. We postulate that TH causes the up-regulation of Apaf-1 and Caspase-9 expression and may activate the intrinsic apoptotic pathway to modulate tumor growth. The possible mechanism demonstrates that TH akin to chemotherapeutic agents may induce apoptosis through multiple signaling pathways that converge on the mitochondria to cause the release of cytochrome c. Cytochrome c binds to Apaf-1 in the presence of dATP/ATP (deoxyadenosine triphosphate/adenosine triphosphate), which then binds to procaspase-9 to form a cytochrome c–Apaf-1–caspase-9 complex, called apoptosome. Apoptosome enables enzymatic self-activation of caspase-9 that subsequently activates procaspase-3. This ultimately results in cell death [51]. Honey mediates cell death mainly through the intrinsic apoptotic pathway and by enhancing pro-apoptotic proteins expression [4, 17].

Our findings showed no evidence of the expression of FASLG and FADD, hence no involvement of caspase-8 or the extrinsic apoptotic pathway in TH mediated apoptosis. Our results are in line with another study which demonstrated that Manuka honey induces intrinsic or caspase-9 apoptotic pathway in breast cancer [52].

A study has reported that over-expression of Bcl-xL in breast cancer patients is associated with metastasis and worse prognosis [53]. The decrease of Bcl-xL expression observed in TH treated tumors suggests that the administration of TH can lead to lower tumor cells proliferation and increased apoptosis by blocking mitochondrial swelling and membrane hyper polarization [54]. Our findings suggest that Bcl-xL expression was hindered by TH at its intrinsic mitochondrial apoptotic pathway. This ultimately promotes apoptosis through increased expression of mitochondrial pathway proteins; Caspase-9 and Apaf-1, as observed in our study. This can be presumed that TH could be a viable option to mediate hematological parameter, and expression of Caspase-9, Apaf-1, E2, Bcl-xL and ESR1 against breast cancer. The variations in the dose dependent effect of TH need further research to elucidate the reasons and the underlying mechanisms.

## Conclusion

Tualang honey when given one week prior to cancer induction and continued for 120 days afterward was found to have significant anti-cancer activity in experimental animal model. It alleviates breast carcinogenesis through modulation of hematologic, estrogenic and apoptotic activities. It also caused slower tumor progression, a lower tumor multiplicity, size and weight, a longer latency period, and better histological features and grading. Tualang honey may be used as a natural ‘cancer-alleviating’ agent or as supplement to chemotherapeutic agents. We believe that current findings could facilitate further research including clinical trials to investigate whether TH could synergize with, or be a substitute for chemotherapeutic drugs.

## Abbreviations

BW: Body weight
IBW: Initial body weight
FBW: Final body weight
ABW: Actual body weight
TH: Tualang honey
RBs: Red blood cells
Hb: Hemoglobin
PCV: Packed cell volume
MCV: Mean corpuscular volume
MCH: Mean corpuscular hemoglobin
MCHC: Mean corpuscular hemoglobin concentration
RDW: Red cell distribution width
Apaf-1: Apoptotic protease activating factor 1
E2: Estradiol
FASLG: Fas ligand
FADD: Fas-associated via death domain
ESR1: Estrogen receptor 1
Bcl-xL: B-cell lymphoma-extra large
-ve control: Untreated group bearing tumor
S.E: Standard error
